# The effects of exergaming on pain, postural control, technology acceptance and flow experience in older people with chronic musculoskeletal pain: a randomised controlled trial

**DOI:** 10.1186/s13102-020-00211-x

**Published:** 2020-10-09

**Authors:** Jae-Llane Ditchburn, Paul van Schaik, John Dixon, Alasdair MacSween, Denis Martin

**Affiliations:** 1 Institute of Science, Natural Resources and Outdoor Studies, University of Cumbria, Fusehill Street, Carlisle, Cumbria, CA1 2HH UK; 2 School of Social Sciences, Humanities and Law, Teesside University, Middlesbrough, TS1 3BA UK; 3 School of Health and Life Sciences, Teesside University, Middlesbrough, TS1 3BX UK

**Keywords:** Exergaming, Exercise therapy, Musculoskeletal Pain, Aged, Aged 80 and over, Postural balance, Heart rate, Flow state experience, Technology acceptance

## Abstract

**Background:**

Older people with chronic musculoskeletal pain are at risk of falls. This study aimed to investigate the effects of exergaming on pain and postural control in older people with chronic musculoskeletal pain. Secondary outcomes were technology acceptance, flow experience, perceived physical exertion, expended mental effort and heart rate.

**Methods:**

Fifty four older adults (age: 71 ± 5 years) with chronic musculoskeletal pain were randomised into 2 groups. Group 1 received exergaming training using the Interactive Rehabilitation and Exercise System (IREX®). Group 2 undertook traditional gym-based exercise (TGB). Both groups completed twice weekly 40-min exercise sessions for 6 weeks. Perceived pain was measured using a numeric pain rating scale and the Multidimensional Affect and Pain Survey questionnaire. Postural control was measured as sway using a Kistler™ force platform. Technology acceptance was measured with the Unified Theory of Acceptance and Use of Technology questionnaire and flow experience with the Flow State Scale. Physiological measures of perceived physical exertion, expended mental effort and heart rate were recorded during all sessions.

**Results:**

The exergaming group demonstrated significant reductions in pain intensity and thermal pain including a near significant approach in physical engagement in comparison to TGB group. Although no intervention effects on postural control were found, the exergaming group showed significant improvements in three sway measures (AP SD, ML SD and AP range) over time whereas significant improvements in ML range were found in the TGB group. Relating to technology acceptance, significant intervention effects on social influence and behavioural intention were found in the TGB group instead, although both groups demonstrated increases of acceptance over time. Regarding flow experience, concentration at task was significantly influenced in the TGB group and significant increases in flow variables over time were observed in both groups. Significant increases over time in perceived physical exertion and expended mental effort were found in both groups.

**Conclusion:**

Our findings support the potential of exergaming to alleviate pain and improve balance in older people with chronic musculoskeletal pain. Both forms of exercise are acceptable, intrinsically motivating and show evidence of benefit to older people with chronic musculoskeletal pain.

**Trial registration:**

ClinicalTrials.gov Identifier: NCT04029285 (retrospectively registered, July 23, 2019)

## Background

Chronic pain is a widespread and debilitating condition; in the UK, in 2017, 34% of adults had chronic pain and in the US, in 2016, 20.4% [1]. Quality of life and health deteriorate, mobility and independence reduce, anxiety and depression increase, as does dependence on medication [2–5]. Pain along with commonly associated symptoms, such as muscle and joint stiffness make moving and exercising difficult [6]. Chronic musculoskeletal conditions, such as low back pain and arthritis also increase the risk of impaired postural control [7–9]. Furthermore, there is evidence to show that older people with two or more body locations of musculoskeletal pain are at risk of falls [10–12].

Unsurprisingly, exercise as a non-surgical, non-pharmacological option is often recommended for older people, especially for those with chronic pain [13, 14] in the hope of increasing activity and independence [15–17] and improving their balance [18–20]. Despite many known benefits, older people are often reluctant to take up exercise, citing individual-level barriers such as fatigue [21], fear the movements will increase their pain [22], or simply a lack interest in exercising [23]. System level barriers such as the lack of infrastructure facilitating exercise for older people also impede uptake amongst them [24, 25].

“Virtual” is defined as something that does not exist physically [26]. When applied to technology, software relevant to these technologies will make the target object appear as if it were physically real [27]. “Virtual reality” is defined as an environment generated by artificial means or computer simulations akin to real-life situations [28, 29]. Exergaming applies digital game technology in a virtual reality environment [30].

Recently, exergaming has been explored as an alternative mode of exercise to encourage physical activity among older people [31–33]. Exergaming systems are currently used for several purposes [34]. Examples of commercial, entertainment based exergaming systems are the Nintendo Wii, Sony PlayStation II and X-box Kinect [35] whereas rehabilitation-specific exergames comprise systems such as the Interactive Rehabilitation and Exercise System (IREX®) [36]. While both types of exergaming systems combine exercise with gaming features, rehabilitation-specific exergames provide feedback on users’ progress, identify impairments and may be personalised [37, 38]. The IREX® [36] uses video capture technology that enables users to see a real-time image of themselves when interacting with the exergames, as opposed to seeing an avatar, as is common in commercial exergaming systems. Whilst commercial exergaming systems have developed and marketed exergames for healthy gamers, the IREX® was designed, developed and adapted with rehabilitation in mind [39, 40]. It provides clinicians with feedback comprising metrics of speed, duration and intensity of workout [36]. Moreover, users do not need to wear, hold or touch anything when playing the exergames [36].

Several exergaming studies report health and wellbeing benefits comparable to those of regular exercise in older people, particularly in balance [41], improvement in age-related kyphosis [42], muscle strength [43], ease of physical movement and psychosocial well-being [44, 45]. In spite of increasing evidence suggesting that that older people are more receptive to using exergaming for exercise [44, 46, 47], majority of exergaming users are young people [48–50]. Gerontology studies have highlighted learned helplessness in older people in using technology [51, 52]. Furthermore, advertising campaigns for exergames tend to target younger age groups with gaming themes that do not appeal to older people [53]. In addition, most studies tested commercially available gaming platforms [43, 54–60] rather than exercise and rehabilitation-specific platforms. Few have studied the effects of exergaming on older people’s chronic musculoskeletal pain and balance using a rehabilitation-specific platform. Furthermore, majority of the studies involving older people used the IREX® [36] to investigate stroke recovery [61–63] and physical rehabilitation [61, 63–66] but none have examined chronic pain and centre of pressure as a measure of postural control in older people with chronic musculoskeletal pain [67].

As such, the primary aim of this study was to assess the effects of exergaming via the IREX® on pain and postural control amongst older people with chronic musculoskeletal pain in comparison with traditional gym-based exercise, with no virtual stimuli (TGB), for older people with chronic musculoskeletal pain. We also wanted to find out if, after having completed an intervention of either exergaming of TGB, our participants found exergaming technology to be acceptable, and whether they experienced flow during the intervention and would consider themselves to continue taking part in that form of exercise. Consequently, secondary aims were to evaluate their technology acceptance, flow state experience and perceived physiological measures during the intervention.

## Methods

### Design

A prospective, randomized, controlled two-arm trial design was used with these groups: (a) exergaming with IREX® and (b) traditional gym-based exercise (TGB). All testing was carried out by the first author who was not blind to participant allocation.

### Setting and participants

Ethical approval was granted by the Teesside University, School of Health and Social Care Research Governance and Ethics Committee, reference number 059/09. The study was conducted in the University’s physiotherapy laboratory.

### Eligibility criteria

Inclusion criteria were male or female, aged 65 years or over, able to walk unassisted (i.e. did not use, or require, any walking aids) for at least 0.5 of a mile and having musculoskeletal pain in two or more joints of more than 12 weeks duration. The inclusion criteria for age follows definitions from Orimo et al. (2006) [68] where 65 years and older constitute “elderly”, equivalent to “older people” in this study, 65 through 74 yeas constitute “early elderly” and over 75 years constitute “late elderly”. Participants with chronic musculoskeletal pain in two or more joints are at risk of falls [10–12]. Therefore participating in this study may elucidate potential benefits for them in terms of balance and improvement in pain.

Exclusion criteria were diagnosis (or suspicion) of any systemic conditions that may cause pain in two or more joints, of more than 12 weeks duration (such as cancer, rheumatic or neurological disease, or condition), self-report of current condition or self-report of history of any condition or injury which would contra-indicate participation in the exercises under study, inability (or any doubt of ability) to give informed consent and inability to read and write English.

### Sample calculation

G*Power version 3.1 [69, 70] was used to conduct a power analysis for a two-group comparison using analysis of covariance to detect a large effect (f = 0.40) for the postural sway outcome measure and 0.80 power. The results showed that the required sample size was 52.

### Recruitment

**Fig. 1:**
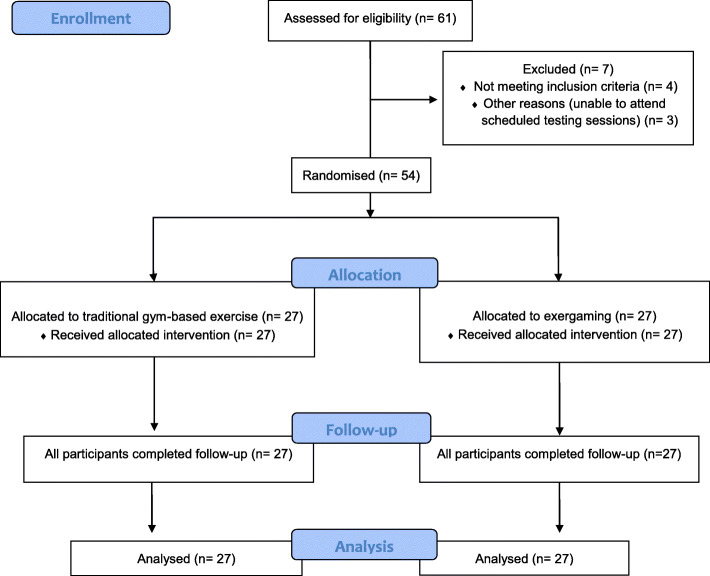
CONSORT flow diagram illustrating recruitment of participants into the study

Participants were recruited from nine local community groups from October to December 2010 (see CONSORT flow diagram, Fig. 1). Sixty-one potential participants were screened for eligibility. Four were excluded due to not meeting the eligibility criteria and three could not attend scheduled sessions. Fifty-four (42 females and 12 males, age: 71 ± 5 years) were allocated to either exergaming with the IREX™ (*n* = 27), or TGB (*n* = 27) (see Table 1). Chronic pain areas were hips, hands/wrists and/or back.

**Table 1:** Participant demographics

	TGB (*n* = 27)	IREX® (*n* = 27)
Male	7	5
Female	20	22
Independent living	26	26
Assisted living	1	1
Retired	26	26
Working part-time	1	1
	Mean (SD)	Mean (SD)
Age (years)	69.78 (4.48)	71.78 (6.10)
Height (cm)	162.16 (6.74)	160.33 (8.60)
Weight (baseline) (kg)	69.27 (13.28)	76. 39 (21.61)
Weight (post) (kg)	68.72 (13.03)	77.31 (22.20)

### Procedure

After written informed consent, demographic information and baseline outcome data were collected and participants were randomly allocated, by stratified blind-card allocation. Appointments for twice weekly, 40 min sessions were arranged for both groups. All exercises were completed on a one-to-one basis, with the first author supervising the sessions (and exercising with the TGB group). All participants began with the same exergames or exercises and progressed at their own pace.

The exergaming group played five IREX® exergames (see Appendix Table 9 for details). Those in the TGB group performed exercises that were matched to the IREX® exergames for movement patterns required, physiological demands, sequence, duration and mode of exercise, by adopting open and closed kinetic chain movements, in the same range and loading, across both groups. Each IREX® exergame was played for 2 minutes and was repeated three times within a session. TGB exercise was conducted in sets of 2 minutes duration and was repeated three times within a session. In both groups participants were given rest periods of 10 to 30 s, or longer, if required, between exergames, or TGB exercise sets.

### Primary outcomes

The primary outcome measures were pain and postural control/sway measured at baseline and after the six-week intervention period. The duration of 6 weeks was selected based on evidence of indications of minimal clinical effects from six-week interventions from previous studies [71].

Pain intensity experienced within 30 days and at present was recorded using a numerical pain rating scale (NPRS) at baseline and after the 6 week intervention period [72]. The NPRS ranges from 0 “no pain” to 10 “worst possible pain” [73, 74]. The sensory, emotional and motivational dimensions of pain were measured using the Multi Affect and Pain Survey (MAPS) questionnaire [75, 76]. MAPS comprises 101 pain descriptors which reflect three major aspects of pain: somatosensory, emotional and well-being. The somatosensory pain supercluster contains 17 clusters with 57 descriptors of painful sensory qualities; the emotional pain supercluster has 8 clusters with 26 descriptors of negative emotional qualities; and the well-being supercluster has 18 descriptors of positive affect, and health, grouped into five clusters. MAPS has been widely validated in pain studies [77–81] and its reliability demonstrated [80].

Postural control was measured as Centre of Pressure (CoP) displacement and velocity, using a portable Kistler™ force platform (Model 9286AA, W 40 x L 60 x H 3.5 cm) with a sampling rate of 1000 Hz [82]. Participants stood barefoot on the Kistler™ force plate and looked directly ahead at a visual target (black 100 mm diameter circle) positioned 3 m from the centre of the force plate at eye level [72, 83, 84] . Participants were asked to stand as still as possible on their dominant leg (preferred kicking), with their eyes open and arms by their side, for three periods of 30s. Between trials, participants stepped off the force plate, to allow calibration of the equipment, which also allowed a 30s rest. This testing sequence was then repeated but with participant’s eyes closed.

### Secondary outcome measures

The secondary outcome measures were technology acceptance, flow experience, perceived levels of physical exertion, subjective mental effort and heart rate, measured at baseline, after each exercise session and after the six-week intervention period. Technology acceptance was measured using the Unified Theory of Acceptance and Use of Technology (UTAUT) [85] questionnaire. The UTAUT comprises a series of 7-point Likert scales ranging from 1 (strongly disagree) to 7 (strongly agree), measuring six domains. The domains are: *performance expectancy* (PE), the degree to which a person believes that using a system will help them attain gains in their performance, *effort expectancy* (EE), the degree of ease in using the technology, *social influence* (SI), the degree to which a person perceives that important others believe they should use the technology, *facilitation conditions* (FC), the degree to which a person believes they should use the technology, *self-efficacy* (SE), the degree to which a person believes they are capable of using the technology and *behavioural intention* (BI), intention to use the intervention again.

Flow experience was measured using the Flow State Scale questionnaire (FSS) [86]. *Flow* is the degree to which people experience an optimal psychological state associated with complete absorption in the task that they are doing (a concept widely researched in various fields) [58, 83, 87, 88]. The FSS consists of 36 questions with nine subscales and response options on a Likert scale from 1 (strongly disagree) to 5 (strongly agree). The subscales are: *autotelic experience* (AE), the intrinsically rewarding experience doing a task, *clear goals* (CG), clearly confident of action, *challenge-skill-balance* (CB), balance between skills and challenge, *concentration at task* (CT), complete control on performing a task, *paradox of control* (PC), at full focus at the task, unambiguous feedback (UF), feedback on performing a task, *action-awareness-merging* (AM), immediate, direct and clear observations whilst performing a task, *transformation of time* (TT), time either speeds up, slows down, becomes irrelevant or out of one’s awareness and *loss of consciousness* (Loss), a sense of not being concerned with oneself while engaging in the activity and in the process; the individual becomes one with the activity, or a part of it.

Perceived levels of physical exertion were measured using the Borg Rating of Perceived Exertion (RPE) scale [89]. Participants subjectively rated their levels of physical intensity and effort based on the physical sensations that they experienced during the exercise session. The scale consists of numbered categories, 6–20 with verbal cues from “very, very light” to “very, very hard”.

Subjective mental effort was measured using the Subjective Mental Effort Questionnaire (SMEQ) (also referred to as the Rating Scale for Mental Effort) [90]. The SMEQ consists of a single scale with nine labels from “Not at all hard to do” to “Tremendously hard to do”.

Heart rate (HR) was recorded using a Polar™ heart rate monitor (FS2C), recording watch and T31 coded chest strap (Polar Electro, Oy, Finland). Mean HR was calculated for each exercise session and recorded as percentage of Age-predicted maximum heart rate (220 - age) (APMHR).

### Exergaming system

Exergaming was performed using five games from the IREX® system (GestureTek, Toronto, Canada), consisting a computer installed with virtual-reality (VR) software, a television monitor with widescreen plasma screen (37″, Hanspree, Type T73B, Netherlands), a digital camera, a green fabric screen (W 3 m x H 2.6 m) and red gloves.

### Postural control data extraction

Range and standard deviation of CoP displacements in the anterior-posterior (AP) and medio-lateral (ML) directions (CoP_AP_ SD, CoP_AP_ range, CoP_ML_ SD, CoP_ML_ all mm) and the resultant CoP velocity (mm.sec^− 1^) were extracted from the force platform using Bioware software (Kistler™), after low-pass filtering of the raw data at 10 Hz. CoP velocity (mm.sec^− **1**^) was calculated using methods described by Raymakers, Samson and Verhaar (2005) [91].

### Statistical analysis

The data were analysed with Version 19 of the Statistical Package for the Social Sciences (SPSS, Chicago, IL, USA). Cronbach’s alpha was computed to assess internal reliability for the subscales of the UTAUT, FSS and MAPS questionnaires, respectively. Analysis of covariance (ANCOVA) was used to assess between-group final scores for each outcome measure used with baseline scores as covariate. Variables that did not meet the assumption of homogeneity of variance were analysed by two-way independent measures ANOVA with blocking using mean splits of scored pre-measures. Mixed analysis of variance (ANOVA) was used to determine any within-subject changes over time. All analyses used a significance level of 0.05. The effect size measure epsilon squared was used, where values of 0.01, 0.06 and 0.14 were interpreted as small, moderate and large [92].

## Results

Participant demographics are shown in Table 1. Descriptive statistics are presented at Table 2. Subscales from the UTAUT [85] and FSS [86] questionnaires demonstrated high internal-consistency reliability exceeding the minimum Cronbach’s alpha of 0.7 [93]. Seventeen clusters from the MAPS questionnaires were deemed reliable having exceeded 0.7. Clusters that did not show internal reliability were temporal pain, faint pain, muscle/joint pain, mental distress, respiratory distress, cold pain, pain restriction, anxiety, emotional avoidance, treatable illness and mentally engaging, with Cronbach’s alpha values ranging from 0.16 to 0.63, respectively.

**Table 2:** Descriptive statistics, mean (SD) for all outcome measures

	Baseline		Post intervention	
	TGB	IREX®	TGB	IREX®
**Primary outcomes**				
Pain intensity				
Experienced within 30 days	6.00 (2.34)	5.52 (2.24)	5.85 (2.43)	5.04 (2.21)
At the time of testing	3.33 (2.82)	2.96 (1.87)	3.48 (3.03)	2.07 (2.11)
MAPS (pain descriptors in parenthesis)				
Somatosensory pain				
Cutaneous (itchy, irritating, crawling, tickling, tingling)	1.13 (1.00)	0.67 (0.53)	1.08 (1.05)	0.63 (0.51)
Autonomic distress (disgusting, nauseating)	0.80 (1.37)	0.09 (0.24)	0.67 (1.35)	0.19 (0.49)
Thermal (burning, hot)	1.41 (1.80)	0.93 (1.30)	1.25 (1.78)	0.56 (0.97)
Pain extent (spreading, persistent, worsening, pervasive)	2.00 (1.52)	1.29 (0.99)	1.80 (1.59)	1.29 (1.03)
Intense pain qualities (vicious, excruciating, nasty, overwhelming)	2.12 (1.79)	0.98 (1.16)	1.91 (1.84)	0.87 (1.20)
Intermittent pressure (throbbing, pounding)	1.46 (1.74)	0.63 (1.11)	1.27 (1.69)	0.71 (1.28)
Brightness (stinging, smarting)	0.56 (1.19)	0.07 (1.18)	0.63 (1.27)	0.19 (0.49)
Incisive pressure (sharp, shooting, biting, deep, tearing, stabbing, gnawing)	1.68 (1.32)	0.20 (0.62)	1.53 (1.28)	0.84 (0.81)
Traction/abrasion (pulling, grinding, squeezing, pressing, cramping, tugging, crushing)	1.12 (1.31)	0.26 (0.70)	0.96 (1.28)	0.54 (0.92)
Numb (numb, numbing)	1.28 (1.84)	0.64 (0.68)	1.13 (1.74)	0.40 (0.92)
Emotional pain				
Physical illness (ailing, suffering)	1.61 (1.38)	0.57 (0.76)	1.38 (1.42)	0.90 (0.88)
Depressed mood (lousy, rejected, depressed, discouraged, miserable, lonely)	0.91 (0.99)	1.35 (1.11)	0.74 (0.90)	0.40 (0.45)
Self-blame (guilty, negligent)	0.48 (0.90)	1.24 (1.12)	0.52 (1.04)	0.40 (0.74)
Anger (angry, outraged, upset, annoyed)	0.83 (1.32)	2.67 (1.36)	0.65 (1.19)	0.43 (0.66)
Fear (alarming. Startling, frantic, terrified)	0.78 (1.34)	0.26 (0.46)	0.69 (1.26)	0.13 (0.36)
Physical avoidance (exhausting, sleepy, tiring, sluggish)	2.14 (1.50)	1.24 (1.12)	1.83 (1.40)	1.13 (1.00)
Well-being				
Physically engaged (active, vigorous)	2.46 (1.65)	2.28 (1.56)	2.69 (1.49)	2.62 (1.42)
Affiliative feelings (loved, forgiving, affectionate, sympathetic)	3.41 (1.44)	2.71 (1.30)	3.57 (1.40)	3.14 (1.40)
Positive affect (hopeful, happy, relaxed, encouraged, cheerful, satisfied, calm)	3.11 (1.51)	2.74 (1.15)	3.32 (1.04)	2.90 (0.94)
Postural sway with eyes open				
AP SD	4.44 (1.40)	5.45 (2.06)	3.92 (1.66)	4.64 (2.03)
AP range	21.42 (5.89)	25.92 (6.25)	18.02 (7.54)	21.25 (6.79)
ML SD	2.13 (0.83)	3.15 (1.89)	1.84 (0.59)	2.56 (1.52)
ML range	12.42 (4.46)	17.82 (10.24)	10.17 (3.78)	13.97 (7.72)
CoP velocity	29.47 (6.72)	32.69 (10.73)	31.48 (10.43)	32.38 (9.58)
Postural sway with eyes closed				
AP SD	4.83 (1.56)	5.45 (1.40)	4.42 (1.79)	5.20 (1.96)
AP range	24.88 (8.12)	28.69 (8.19)	21.24 (8.29)	27.70 (9.17)
ML SD	2.27 (1.31)	2.62 (1.45)	1.95 (0.83)	2.32 (0.78)
ML range	14.45 (9.27)	15.06 (7.76)	10.86 (4.02)	12.92 (4.38)
CoP velocity	30.69 (8.27)	37.32 (9.91)	30.83 (10.40)	33.89 (10.16)
**Secondary outcomes**				
UTAUT				
Performance expectancy	4.16 (2.22)	3.54 (1.56)	6.67 (0.48)	6.13 (1.09)
Effort expectancy	4.04 (1.95)	3.23 (1.46)	6.26 (0.82)	5.70 (1.16)
Social influence	3.54 (2.41)	3.19 (1.71)	6.13 (1.28)	4.70 (1.84)
Facilitating conditions	4.08 (2.12)	3.77 (1.81)	6.21 (0.91)	5.56 (1.29)
Self-efficacy	3.70 (1.93)	3.17 (1.52)	5.90 (1.05)	5.22 (1.46)
Behavioural intention	3.55 (2.11)	2.88 (1.99)	6.58 (0.68)	5.85 (1.47)
FSS				
Autotelic experience	3.00 (1.43)	3.41 (1.28)	4.16 (0.54)	4.10 (0.80)
Clear goals	3.05 (1.27)	2.92 (1.25)	4.53 (0.46)	4.36 (0.76)
Concentration at task	2.96 (1.22)	3.31 (1.26)	4.53 (0.44)	4.31 (0.74)
Paradox of control	2.82 (1.36)	2.84 (1.24)	4.40 (0.66)	4.08 (1.01)
Challenge-skill-balance	2.93 (1.06)	3.04 (1.01)	4.42 (0.51)	4.04 (0.76)
Unambiguous feedback	2.81 (1.26)	2.91 (1.12)	4.41 (0.62)	4.21 (0.76)
Action-awareness-merging	2.46 (1.03)	2.67 (1.01)	4.09 (1.02)	3.89 (0.84)
Transformation of time	2.55 (1.16)	3.05 (1.21)	3.75 (1.28)	3.56 (1.19)
Loss of self-consciousness	3.09 (1.42)	3.31 (1.25)	4.52 (0.56)	4.40 (0.74)
Objective and subjective measures of physiological demand				
Perceived physical effort (RPE)	10.48 (1.85)	9.41 (1.31)	10.77 (1.65)	9.81 (2.07)
Subjective mental effort	39.47 (11.57)	32.46 (9.95)	55.93 (15.70)	40.96 (16.28)
Heart rate	77.41 (5.69)	77.67 (4.45)	82.23 (11.00)	81.80 (9.58)

### Primary outcomes

#### Pain intensity

No effect of intervention was found on self-reported pain intensity experienced within 30 days before and after the intervention, and pain intensity at the time of testing, as determined by the ANCOVA with pre-measures as the covariate (see Table 3). Although the mixed ANOVA did not show any significant differences in pain intensity over time, the interaction effect between time and intervention was significant for pain intensity experienced at the time of testing in favour of exergaming (F [1. 52] = 3.98, *p* = 0.05, *ε*^*2*^ *=* 0.46, large effect). The 30% significant reduction in perceived pain intensity in the exergaming exceeded the appropriate cut-off point for determining the minimal clinically important differences (MCID) of changes in pain intensity of 15%, where a numerical rating change score of − 2.0 and a percent change score of − 33% are best associated with the concept of “much better improvement” [94].

**Table 3:** Adjusted post-intervention between group difference (ANCOVA) and within-group change over time (mixed ANOVA); Mean differences (95% CI) for both measures of pain intensity

Outcome	Adjusted post-intervention difference between groups (ANCOVA)	Within-group change over time (mixed ANOVA)	
	IREX® - TGB	IREX®	TGB
Overall pain intensity experienced within 30 days before and after the intervention	− 0.45 (−1.25 to 0.36)	− 0.48 (−1.30 to 0.34)	− 0.15 (− 0.51 to 0.21)
Pain intensity experienced at baseline and after the intervention	−1.12 (−2.15 to − 0.09)	− 0.89** (− 1.52 to − 0.26)	0.15 (− 0.71 to 1.01)

**p < 0.01

### Multidimensional affect and pain variables (MAPS)

ANCOVA revealed that the variable physically engaged; (F [1, 48] = 3.76, *p* = 0.06, *ε*^*2*^ *=* 0.01, small effect) from the well-being subcluster approached significance in favour of exergaming (see Table 4). This suggests meaningful increases in older people’s feelings of being active and vigorous after exergaming. Thermal pain (F [1, 48] = 14.43, *p* = 0.00, *ε*^*2*^ *=* 0.09, medium effect) showed a significant effect of intervention in favour of exergaming.

**Table 4:** Adjusted post-intervention between group difference (ANCOVA) and within-group change over time mean differences (95% CI) for Multidimensional Affect and Pain Survey (MAPs) measures

MAPS	Adjusted post-intervention difference between groups (ANCOVA)	Within-group change over time (mixed ANOVA)	
	IREX® – TGB	IREX®	TGB
**Somatosensory pain supercluster**			
Cutaneous	−0.34 (− 0.22 to 0.15)	− 0.06 (− 0.16 to 0.03)	−0.06 (− 0.21 to 0.09)
Autonomic distress	0.17 (− 0.15 to 0.49)	0.10 (− 0.08 to 0.27)	− 0.10 (− 0.24 to 0.06)
Thermal^a^	−1.06*** (− 1.62 to − 0.50)	−0.38 (− 0.82 to 0.05)	−0.08 (− 0.29 to 0.13)
Pain extent	− 0.42 (− 0.85 to − 0.04)	−0.05 (− 0.34 to 0.25)	−0.17 (− 0.42 to 0.07)
Intense pain qualities	− 0.15 (− 0.71 to 0.40)	−0.12 (− 0.49 to 0.26)	−0.20 (− 0.59 to 0.19)
Intermittent pressure	0.04 (− 0.59 to 0.68)	0.12 (− 0.38 to 0.61)	−0.23 (− 0.66 to 0.20)
Brightness	0.10 (− 0.32 to 0.51)	0.12 (− 0.06 to 0.29)	0.08 (− 0.18 to 0.33)
Incisive pressure	−0.09 (− 0.53 to 0.34)	−0.06 (− 0.30 to 0.19)	−0.12 (− 0.46 to 0.22)
Traction/abrasion	0.16 (− 0.23 to 0.55)	0.02 (− 0.22 to 0.26)	−0.14 (− 0.44 to 0.16)
Numb	0.15 (− 0.37 to 0.66)	0.13 (− 0.15 to 0.42)	−0.19 (− 0.57 to 0.18)
**Emotional pain supercluster**			
Physical illness^a^	−0.13 (− 0.74 to 0.49)	−0.12 (− 0.44 to 0.20)	−0.15 (− 0.37 to 0.06)
Depressed mood^a^	− 0.57 (− 1.23 to 0.09)	−0.22* (− 0.39 to − 0.05)	−0.10 (− 0.23 to 0.04)
Self-blame	− 0.02 (− 0.23 to 0.19)	0.00 (− 0.10 to 0.10)	0.02 (− 0.18 to 0.21)
Anger	−0.06 (− 0.37 to 0.25)	−0.16 (− 0.38 to 0.05)	−0.17 (− 0.46 to 0.11)
Fear	− 0.21 (− 0.53 to 0.11)	−0.13 (− 0.28 to 0.01)	−0.12 (− 0.41 to 0.18)
Physical avoidance	− 0.13 (0.56 to 0.31)	−0.14 (− 0.43 to 0.15)	−0.21 (− 0.56 to 0.14)
**Well-being supercluster**			
Physically engaged^+^	0.13^+^ (−0.29 to 0.56)	0.33 (−0.13 to 0.79)	0.13 (− 0.02 to 0.29)
Affiliative feelings	0.16 (−0.27 to 0.59)	0.42^++^ (− 0.004 to 0.85)	0.14* (0.02 to 0.27)
Positive affect	−0.16 (− 0.56 to 0.24)	0.17 (− 0.21 to 0.54)	0.11 (− 0.26 to 0.48)

^a^ Variable that has violated homogeneity of regression for ANCOVA

^+^ Approaching significance, *p* < 0.10

^++^ *p* = 0.05

*p < 0.05, ***p < 0.001

The mixed ANOVA revealed significant effects of time on depressed mood (F [1, 50] = 9.09, *p* = 0.004, *ε*^*2*^ *=* 0.01, small effect) and affiliative feelings (F [1, 50] = 6.92, *p* = 0.01, *ε*^*2*^ *=* 0.03, small effect) in favour of exergaming. Moreover, three variables approached significance also in favour of exergaming. They were thermal pain (F [1, 50] = 3.85, *p* = 0.06, *ε*^*2*^ *=* 0.01, small effect), anger (F [1, 50] = 3.76, *p* = 0.06, *ε*^*2*^ *=* 0.01, small effect) and physically engaged (F [1, 50] = 3.82, *p* = 0.06, *ε*^*2*^ *=* 0.01, small effect).

### Postural control

Although the ANCOVA did not reveal any effect of intervention on postural control, the mixed ANOVA showed that there were significant reductions over time for AP SD (F [1, 46]= 8.29, *p* = 0.01, *ε*^*2*^ *=* 0.09, medium effect), ML SD (F [1, 46]= 8.37 *p* = 0.01, *ε*^*2*^ *=* 0.05, nearly medium effect), AP range (F [1, 45]= 9.91, *p* = 0.003, *ε*^*2*^ *=* 0.16, large effect) and ML range (F [1, 45]= 4.12, *p* = 0.05, *ε*^*2*^ *=* 0.06, medium effect) during bipedal standing with vision, and for CoP excursion in the medio-lateral direction (F [1, 47]= 5.43, *p* = 0.03, *ε*^*2*^ *=* 0.08, medium effect) during pedal standing without vision (see Table 5).

**Table 5:** Adjusted post-intervention between group difference (ANCOVA) and within-group change over time mean differences (95% CI) for postural control

Postural control	Adjusted post-intervention difference between groups (ANCOVA)	Within-group change over time (mixed ANOVA)	
	IREX® – TGB	IREX®	TGB
Bipedal – eyes open			
AP SD	0.32 (−0.63 to 1.28)	0.84* (0.20 to 1.48)	0.62 (−0.23 to 1.47)
ML SD	0.19 (−0.33 to 0.71)	0.58* (1.06 to 0.00)	0.24 (−0.06 to 0.54)
CoP velocity	−1.10 (−6.00 to 3.77)	−0.36 (− 3.41 to 2.69)	− 2.33(−6.45 to 1.79)
AP range	1.70 (− 2.54 to 5.94)	4.58* (1.14 to 8.02)	2.88 (− 0.62 to 6.38)
ML range	1.94 (− 1.94 to 5.82)	3.74 (− 1.04 to 8.53)	1.37 (− 0.46 to 3.19)
Bipedal – eyes closed			
AP SD	0.52 (−0.48 to 1.52)	0.49 (− 0.64 to 1.62)	0.15 (− 0.31 to 0.62)
ML SD	0.29 (− 0.11 to 0.70)	0.19 (− 0.28 to 0.66)	0.32 (− 0.16 to 0.79)
CoP velocity	0.11 (− 5.63 to 5.84)	2.31 (− 1.10 to 5.72)	− 0.56 (− 5.56 to 4.45)
AP range	4.10 (− 0.90 to 9.09)	0.08 (−3.44 to 3.61)	2.85 (− 1.09 to 6.79)
ML range	2.13 (− 0.13 to 4.40)	1.32 (− 1.14 to 3.79)	3.72^+^ (− 0.05 to 7.48)

*p < 0.05

^+^*p* = 0.05

### Technology acceptance

UTAUT scores increased in both groups which indicated moderate-to-high acceptance for both exergaming and TGB. The ANCOVA found significant effects of intervention in favour of TGB for social influence (F [1, 44] = 5.16, *p* = 0.03, *ε*^*2*^ = 0.06, medium effect) and behavioural intention (F [1, 44] = 4.99, *p* = 0.03, *ε*^*2*^ *=* 0.08, medium effect) (see Table 6). Higher mean values occurred in the control group indicating a greater level of acceptance towards TGB rather than exergaming.

**Table 6:** Adjusted post-intervention between group difference (ANCOVA) and within-group change over time mean differences (95% CI) for technology acceptance

UTAUT	Adjusted post-intervention difference between groups (ANCOVA)	Within-group change over time (mixed ANOVA)	
	IREX® – TGB	IREX®	TGB
Performance expectancy	−0.55 (− 1.04 to − 0.05)	1.40** (0.67 to 2.13)	2.14*** (1.32 to 2.96)
Effort expectancy	− 0.48 (− 1.04 to 0.08)	1.49*** (0.88 to 2.10)	1.80*** (1.04 to 2.65)
Social Influence	−1.39* (− 2.24 to − 0.54)	1.06** (0.30 to 1.82)	2.46*** (1.65 to 3.27)
Facilitating conditions	−0.66 (− 1.31 to − 0.01)	1.02* (0.23 to 1.80)	1.98*** (1.10 to 2.85)
Self-efficacy	− 0.63 (− 1.39 to 0.13)	1.22* (0.28 to 2.16)	1.89*** (1.08 to 2.70)
Behavioural intention	−0.69* (− 1.35 to − 0.03)	1.65*** (0.88 to 2.43)	2.17*** (1.26 to 3.09)

*p < 0.05, **p < 0.01, ***p < 0.001

The mixed ANOVA revealed a statistically significant increases over time for all the UTAUT measures – performance expectancy (F [1, 46] = 45.04, *p* < 0.001, *ε*^*2*^ = 0.36, large effect), effort expectancy (F [1, 46] = 49.40, *p* < 0.001, *ε*^*2*^ = 0.37, large effect), social influence (F [1, 46] = 42.69, *p* < 0.001, *ε*^*2*^ = 0.34, large effect), facilitating conditions (F [1, 46] = 28.07, *p* < 0.001, *ε*^*2*^ = 0.27, large effect), self-efficacy (F [1, 46] = 26.27, *p* < 0.001, *ε*^*2*^ = 0.27, large effect) and behavioural intention (F [1, 46] = 43.96, *p* < 0.001, *ε*^*2*^ = 0.38, large effect). A significant interaction effect between time and intervention was found for social influence (F [1, 46] = 6.73, *p* = 0.01, *ε*^*2*^ = 0.05, almost medium effect) in favour of TGB.

### Flow

The ANCOVA (shown in Table 7) revealed a significant effect of intervention on concentration of task (F [1, 44] = 5.67, *p* = 0.02, *ε*^*2*^ *=* 0.09, medium effect) favouring TGB whereas autotelic experience (F [1, 44] = 4.06, *p* = 0.05, *ε*^*2*^ *=* 0.04, small effect) and paradox of control (F [1, 44] = 3.63, *p* = 0.06, *ε*^*2*^ *=* 0.05, medium effect) approached significance, also in favour of TGB. Nevertheless, the results showed a direction of increase in post-intervention scores for these variables. No effect of intervention was found on the other variables: challenge-skill-balance (F [1, 44] = 3.21, *p* = 0.08, *ε*^*2*^ *=* 0.04), transformation of time (F [1, 44] = 2.09, *p* = 0.16, *ε*^*2*^ *=* 0.02), loss of consciousness (f [1, 44] = 1.29, *p* = 0.26, *ε*^*2*^ *=* 0.01), feedback (F [1, 44] = 1.96, *p* = 0.17, *ε*^*2*^ *=* 0.02). The same was found for variables that did not meet homogeneity of regression as determined by mixed ANOVA by blocking: clear goals (F [1, 44] = 1.25, *p* = 0.27, *ε*^*2*^ *=* 0.01) and action-awareness-merging (F [1, 44] = 0.47, *p* = 0.50, *ε*^*2*^ *=* 0.00).

**Table 7:** Adjusted post-intervention between group difference (ANCOVA) and within-group change over time mean differences (95% CI) for flow

FSS	Adjusted post-intervention difference between groups (ANCOVA)	Within-group change over time (mixed ANOVA)	
	IREX® – TGB	IREX®	TGB
Autotelic experience	−0.16^+^ (− 0.47 to 0.15)	0.79*** (0.40 to 1.18)	1.16*** (0.65 to 1.68)
Clear goals^a^	−0.07 (− 0.43 to 0.30)	1.44*** (0.97 to 1.9)	0.93 (− 0.36 to 2.23)
Challenge-skill-balance	−0.36 (− 0.73 to 0.02)	1.06*** (0.61 to 1.51)	1.46*** (0.94 to 1.98)
Concentration at task	−0.24* (− 0.59 to 0.11)	1.09*** (0.59 to 1.59)	1.54*** (0.94 to 2.15)
Paradox of control	−0.30^+^ (− 0.79 to 0.19)	1.33*** (0.79 to 1.87)	1.49*** (0.83 to 2.15)
Unambiguous feedback	−0.20 (− 0.60 to 0.21)	1.38*** (0.91 to 1.85)	1.52*** (0.93 to 2.12)
Action-awareness-merging^a^	−0.20 (− 0.73 to 0.32)	1.28*** (0.87 to 1.69)	1.55*** (0.87 to 2.24)
Transformation of time	−0.72 (−2.05 to 0.60)	0.55* (0.08 to 1.02)	1.25 (−0.33 to 2.83)
Loss of self-consciousness	−0.14 (− 0.52 to 0.25)	1.15*** (0.63 to 1.67)	1.40*** (0.76 to 2.04)

^a^ Variable that has violated homogeneity of regression

^+^ Approaching significance, *p* < 0.10

*p < 0.05, ***p < 0.001

Mixed ANOVA found significant increases over time in all of the flow variables – autotelic experience (F [1, 46] = 40.20, *p* < 0.001, *ε*^*2*^ = 0.23, large effect), clear goals (F [1, 46] = 13.57, *p* < 0.001, *ε*^*2*^ = 0.16, large effect), challenge-skill-balance (F [1, 46] = 57.69, *p* < 0.001, *ε*^*2*^ = 0.32, large effect), concentration at task (F [1, 46] = 49.27, *p* < 0.001, *ε*^*2*^ = 0.32, large effect), paradox of control (F [1, 46] = 47.46, *p* < 0.001, *ε*^*2*^ = 0.33, large effect), unambiguous feedback (F [1, 46] = 63.12, *p* < 0.001, *ε*^*2*^ = 0.37, large effect), loss of consciousness (F [1, 46] = 56.01, *p* < 0.001, *ε*^*2*^ = 0.35, large effect), transformation of time (F [1, 46] = 21.96, *p* < 0.001, *ε*^*2*^ = 0.16, large effect) and loss of self-consciousness (F [1, 46] = 41.39, *p* < 0.001, *ε*^*2*^ = 0.29, large effect).

### Physiological outcomes

The ANCOVA did not reveal significant effect of interventions on the physiological measures, although heart rate was higher in the exergaming group. The mixed ANOVA however, revealed significant increases over time in perceived physical effort (F [1, 46] = 11.24, *p* = 0.002, *ε*^*2*^ *=* 0.07, medium effect) and perceived subjective mental effort (F [1, 46] = 15.12, *p* = 0.00, *ε*^*2*^ *=* 0.09, medium effect) (see Table 8). Post-intervention scores for perceived physical exertion and mental effort were lower in the exergaming group compared to the control group (as earlier shown in Table 2). These scores suggest that exergaming may be less strenuous and require less effort compared to TGB.

**Table 8:** Adjusted post-intervention between group difference (ANCOVA) and within-group change over time mean differences (95% CI) for objective and subjective measures of physiological demand

	Adjusted post-intervention difference between groups (ANCOVA)	Within-group change over time (mixed ANOVA)	
	IREX® – TGB	IREX®	TGB
Rating of Perceived Exertion	0.29 (−0.68 to − 1.25)	0.99^+^ (− 2.04 to 0.06)	− 1.00** (− 1.57 to − 0.43)
Subjective Mental Effort	5.69 (− 4.01 to 15.38)	−14.24*** (− 22.96 to − 5.51)	−5.80* (− 11.56 to − 0.03)
% of Age Predicted Maximal Heart Rate^a^	1.43 (− 1.41 to 4.27)	−0.88 (− 3.02 to 1.26)	0.42 (−1.98 to 2.82)

^a^ Variable that has violated homogeneity of regression

^+^ Approaching significance, *p* < 0.10

*p < 0.05, **p < 0.01, ***p < 0.001

## Discussion

The primary aim of this present study was to investigate the effects of exergaming on pain and postural control amongst older people with chronic musculoskeletal pain. Exergaming with the IREX® [36] was compared with TGB in two groups. Overall, results suggested that exergaming was beneficial in terms of ameliorating pain and improving balance in older people in this population. The secondary aim of the study was to investigate technology acceptance and flow experience of the intervention including perceived physiological outcomes, enabling us to understand if older people with chronic musculoskeletal pain would find exergaming (or TGB) to be acceptable and considered themselves willing to use it. Henceforth, our results showed that older people with chronic musculoskeletal pain were receptive to exergaming and TGB, and had experienced flow in both forms of exercise.

### Pain

Despite evidence of therapeutic benefits from exergaming [43, 95, 96], published studies on the effects of exergaming on pain are varied and inconsistent [97]. Many suggest an association between exergaming and pain [98] but few report significant changes in pain after exergaming [98, 99]. Kim et al. (2014) [100] found significant improvements in the Oswestry low-back pain disability index (ODI) scores amongst middle-aged women with low back pain after a three-times weekly 4-week exergaming intervention using Wii Fit Yoga. Sobral Monteiro-Junior et al. [98] found significant reductions in chronic low back pain amongst older women after a three-times weekly 8-week using both exergaming and strength exercises, but failed to find an intervention effect. According to Witmer and Singer [101], higher levels of presence in users may be attained by a virtual environment that produces a greater sense of immersion. When interacting with the exergame, the user becomes immersed in the virtual world. Hence, their attention shifts from their natural state of being. In our study, perceived pain intensity when tested at the end of the intervention was significantly reduced in favour of exergaming despite the prevalence of chronic pain throughout the six-week intervention (as shown earlier in Table 2). Our results broadly support the view that the virtual reality aspect used in exergaming may alter pain perception to some extent through active distraction [102, 103].

We are the first to use the MAPS questionnaire across exergaming and standard exercise. In terms of the multidimensional aspects of pain, we observed significant improvement in thermal pain (pain related to heat sensations) and feelings of physical engagement (active, vigorous) in the exergaming group. This suggests that exergaming may have alleviated the experience of pain to some extent [104]. Over time, significant improvements in depressed mood and affiliative feelings were also in favour of exergaming. While the control group also showed improvements in depressed mood over time, the reduction was significantly higher in the exergaming group. This suggests meaningful increases of older people’s feelings of being active and vigorous and benefits in emotional well-being after exergaming. Our findings agree with the premise that exergaming may induce positive mood states in users [105, 106].

### Postural control

In our study, we did not find significant post-intervention differences between the exergaming and TGB groups for both conditions of eyes open and eyes closed. However, our findings show improvements in balance similar to those reported by Sobral Monteiro-Junior et al. [98] and Bisson et al. [107], where we identified significant within-groups differences over time for some postural sway measures as determined by mixed ANOVA. While of the postural control measures decreased over time in both groups with eyes open and closed, statistically significant reductions over time were observed on ML SD, AP SD and the CoP excursion in the anterior-posterior and medio-lateral direction in the exergaming group with vision, indicating better postural control. These improvements in balance are encouraging in comparison with those reported by Barry et al. [83]. They found significant improvements over time in ML SD, ML range and CoP velocity in healthy adults who had participated in a three-times weekly exergaming intervention for 4 weeks. Our findings are also consistent with those of Whyatt et al. [108] who found significant increases in Berg Balance Scale (BBS) scores, higher balance confidence and increased performance in levels of CoP displacement in the anterior, right and left CoP test locations after exergaming.

We also found significant reductions over time observed in the CoP excursion in the medio-lateral direction for the TGB group under eyes closed condition similar to those of Nicholson et al. [109], who reported significant reductions in medio-lateral CoP range in older people following 12 weeks of balance training. ML postural sway is associated more with fall risks in older people compared to AL postural sway [110]. The effect of exercise on postural control becomes more apparent when the balance task is performed without vision [111]. When the eyes are closed, balance relies solely on efferent neuromuscular and sensorimotor input [112], which can be improved with exercise [113]. Our finding reinforces the premise that TGB exercise (in this case), has the potential to improve balance in older people when performed safely. There were no adverse events, reactions or report of motion sickness amongst participants in our study. Both forms of exercise appeared to yield some benefit. Although we are unable to confirm the postural control mechanisms that were improved with exergaming in this study, our results reflect the underlying positive effect of exergaming on postural control. We suggest that exercising using exergames can potentially contribute to improving balance and reducing fall risks in older people with chronic musculoskeletal pain [111].

### Technology acceptance

Our results showed that all UTAUT scores increased in both groups but significance was achieved only in social influence and behavioural intention in the TGB exercise group. We speculate that the increase in all UTAUT scores indicated high acceptance for both forms of exercise and favourable response from participants in both groups. This could be due to several factors. Firstly, the affective state of a user plays an important role in their acceptance of a new activity or technology [114]. How users feel when they perform the exercises determines their appraisal of the exercise and whether they would continue with it [115]. Kwan and Bryan [116] found that affective response influenced exercise behaviour, particularly intention to exercise. In the case of exergaming, Billis et al. [31] found that game content in exergames adapted according to older people’s affective states would influence their acceptability to exergaming. Secondly, if older people found the type of exercise to be both useful and easy to follow, they were more likely to express intention to continue the activity [117]. Thirdly, verbal or non-verbal social behaviour nurtures change in any particular behaviour [118, 119]. This would include encouragement, feedback or supervision and even the mere presence of the researcher during the sessions [120].

Our results indicated high acceptance for both forms of exercise and favourable response from participants in both groups. However, significance was observed only in social influence and behavioural intention in the TGB group. Several factors could have influenced this increase. The standard exercise movements did not involve complex movements or high physical intensities [121]. The higher scores in effort expectancy in the standard exercise group is presumably because the movements did not involve interaction with an external source. The TGB exercise routine comprised planned and structured repetitive physical movements [122]. Therefore, the participants were exercising with themselves instead of having to engage with visual or auditory stimuli (as in exergaming). This could have made the exercising process easier. We also observed that the change in behavioural intention was larger in the exergaming group although it did not reach significance. We speculate that our sample experienced positive affect and engagement during exergaming, which could have brought on the larger increase in behavioural intention [123]. This would require verification with a larger-scale study.

### Flow

We found significant between-group differences in the concentration aspect of flow state, favouring the TGB group. Two other dimensions, autotelic experience, and paradox of control approached significance, also favouring the TGB group. While our results showed a trend of increased scores in all flow dimensions from baseline to the end of the intervention, significance increases over time were achieved in eight of the nine dimensions of flow state in both groups, except transformation of time, supporting the notion of the flow phenomenon in sport [124, 125].

Similar results have been reported in previous studies [58, 83], which suggests that the immersive environment during exergaming can facilitate distortion of time amongst users. Distortion of time during exercise implies that users experience deep involvement when exercising and become fully invested in the exercise experience [126]. The largest effect size demonstrated in the significant increase in unambiguous feedback in the exergaming group suggests that the exergaming group received more direct and immediate feedback when exercising in an immersive environment compared to performing standard exercises. This feedback is akin to successes and failures when playing the exergames so that a clear idea and continuity of feedback is provided for the next action [127].

### Physiological measures

Significant increases over time in perceived physical exertion and expended subjective mental effort in both groups suggested that our participants invested more physical effort and concentration into their respective exercise sessions. In the comparison of post-intervention scores between the two groups, scores for perceived physical exertion and mental effort were lower in the exergaming group. Barry et al. [83] also reported significantly lower post-intervention physical exertion scores in their study comparing Xbox Kinect™ with traditional gym-based exercise. The higher scores in expended mental effort in our study reflect the role of cognition in performing motor skills required in physical activity [128]. Where thought processes involved in exercise tends to get easier with practice [129], more mental effort invested seen in our study could be due to a factor of time because it was a short-term study for participants to learn the movements.

While both groups recorded increases in heart rate over time, heart rate values were slightly higher in the exergaming group. This is speculative of higher physical intensity when exercising with the exergames [130]. Nevertheless, the lack of significant differences in heart rate for either group at 77% of APMHR places exercise intensity in both groups within the *Vigorous* classification (77–95% of HR Max) of the ACSM [131] even though the participants RPE levels (of around 10) is associated with light exercise. Interestingly, this apparent anomaly suggests that participants may have underrated their exertion levels compared to normative values and expectations for RPE. It also appears that while both groups were exercising at a high aerobic physiological demand, which did not alter (as reflected by % of APMHR), participants felt the exercise was somewhat easier throughout, and, despite the perception of effort increasing over the intervention period, their RPE remained below levels normally associated with vigorous exercise (RPE 14–17) [131].

We did not find evidence of significant post-intervention differences in perceived physical exertion, expended subjective mental effort and heart rate between the exergaming group and the control group. Our results show that required movements for the exercises were successfully matched and hence any differences mentioned earlier between the groups could also be attributed to the different exercises they undertook. Future research could include exergaming intensity at different levels to evaluate physiological effects in older people with chronic musculoskeletal pain.

### Limitations

We acknowledge that our results are based on a limited number of participants. As such, the study was not sufficiently powered to generate fully definitive results for the other comparisons (for example, some of the MAPS subclusters etc.). There was no follow up to evaluate long term effects amongst participants. As this research was conducted as part of the completion of a PhD, it was also restricted by staffing, time and funding. For practical reasons, neither the researcher nor the participants were blind to the conditions being tested. In future, this research would benefit from further verification from a larger sample.

## Conclusion

Exergaming was found to be comparable to standard exercise in terms of acceptance and its effects on pain and postural control, in addition to flow experience. This could be potentially attributed to its aspect of virtual reality. Our study shows that older people with chronic musculoskeletal pain could benefit from at least subtle improvements in balance after taking part in short-term exergaming. Although significantly higher post-intervention flow state scores were found in the standard exercise group, there was no evidence to show absence of flow experience in exergaming. In exergaming programmes run by certain healthcare or rehabilitation centres, clients are supported by their physiotherapist in terms of setting suitable exergaming levels for them, monitoring and prescribing rehabilitative movements for them [132, 133]. With this, another consideration is the potential advantage that after initial instruction, exergaming may require less supervision by physiotherapy staff and rely on continuous direct feedback to each patient. This implies that users may gradually become more independent in performing their prescribed body movements into game-play. Overall, our findings suggest that exergaming is potentially effective and may be suitable for older people with chronic musculoskeletal pain.

### Future directions

Future research could include a follow-up to assess the duration of any effects, investigate gender differences in pain and balance outcomes and evaluate exergaming without supervision. Postural control mechanisms could also be evaluated in depth. In addition, the gap in the literature regarding the lack of minimally clinically difference in postural control measures via CoP could also be addressed. This research could be extended to include using commercial exergaming technology such as the Sony Playstation®4 [134], Nintendo Wii Plus and selected exergames in Nintendo Switch [135]. Further work could also evaluate the effects of exergaming in a sample of older people who are affected to a greater extent of chronic musculoskeletal pain and hence, are more disabled than of those in this study.

## Data Availability

The datasets during and/or analysed during the current study are available from the corresponding author on reasonable request.

## Appendix

**Table 9:** Comparison of exercises undertaken by the traditional gym based exercise group (TGB) and the exergaming (IREX®) group

Games	TGB	IREX™	Movements required
Volleyball	Stand up straight with knees slightly bend and your feet shoulder width apart. Clasp both hands in front of your abdomen and slowly raise both arms to the front until eye level, and lower both arms. Repeat three times. Following this, stand comfortably with both arms by your side. Raise the right arm away from your body until shoulder level and then lower it down again to your side. Repeat with the left arm. Following this, move two steps to the right and repeat the movement of the arms; repeat with movement to the left.	Land the ball in your opponent’s court or outside your court. Either move your body, shoulder or touch the volleyball by hand. Smoother movements allow better contact with the ball.	Full medial and lateral weight shifting. Vertical stretches, moving the upper extremities and whole body movement.
Sharkbait	Stand up straight with knees slightly bent and your feet at a comfortable width apart. Stretch out both arms so that they form a T with your body and slowly bend your knees to a comfortable position. Keep your back straight, while in this position, transfer your weight to the right leg and reach out to the right side with your upper torso and right arm as much as you can. Hold for 2 s and gently move your position back as you were before you reached to the right. Repeat with the left side.	You will see yourself virtually deep-sea diving with sea creatures. Catch as many stars as you can. Lean side-by-side, crouch down or raising your arms. To move sideways quickly, step to the side. If you meet a shark, it will virtually swallow you and expel you out of its mouth. Contact with an electric eel virtually temporarily disables your movement.	Full medial and lateral weight shifting of the centre of gravity body movement with bending and stretching.
Formula racing	Stand up straight with knees slightly bent and your feet shoulder width apart. Gently hold both hands in front of your torso with both elbows bent. Look straight ahead while maintaining a relaxed stance, and gently turn your body to the right and back to original position, then to the left and back to original position. Repeat this time with your arms extended.	You will see yourself virtually driving in a Grand Prix. The course of the track is also visible to you. Drive through the racecourse as best as you can. Steer by stepping to the right or left, by moving your body to the side, or by moving one arm at a time. If you feel that you have not moved on the track, take one small step to the side to move your car.	Full medial and lateral weight shifting of the centre of gravity body movement by bending and stretching. Left and right trunk movements and movement of the upper torso.
Snowboard	Stand up straight with your feet shoulder width apart. Place your hands in front of your body as if to hold an imaginary ball and look straight ahead. Move your pelvis to the front (towards your hands) and hold for 2 to 3 s, and to the back. Re-peat as many times as you can. Next stand upright and take a comfortable step forward with your right foot (almost into a lunge position). Rest your hands on your hips and gently tilt your body to the right and back to where you started. Repeat this by standing upright again, this time with a step forward with your left foot, resting your hands on your hips and gently tilting your body to the left, and back to where you started. Try to keep your upper body upright and your back as straight as possible.	You will see a red silhouette of yourself standing on a snowboard, coming down a narrow slope, and a virtual image of yourself when you cross the finish line. Begin by stepping sideways until you are centred over the snowboard. Make as many jumps as possible and avoid hitting other objects. Lean to either side, or move your arm to one side.	Full medial and lateral weight shifting of the centre of gravity body. Vertical movements, pelvic tilt and movement for hamstrings.
Birds and balls	Stand up straight with your feet shoulder width apart. Place both arms at your sides. Beginning with the right arm: slowly move your right arm upwards until shoulder level and gently open and close your right hand (this involves movement of the thumb, fingers and palm). Repeat with your left arm. As you progress through the sessions, use both arms at different positions (e.g. to the top of your head, stretching to the top left or right).	You will virtually be in a pastoral background with colourful balls coming at you. Touch the balls with any part of your body e.g. once you have exercised with your right shoulder or arm, you may repeat it with the left.	Anterior and medial-lateral weight shifting of the centre of gravity over base of support. Shoulder rotation and flexion and movement of the upper extremities

## Abbreviations

AE: Autotelic experience
AM: Action-awareness-merging
APMHR: Age-predicted maximum heart rate (220 - age)
AP: Anterior-posterior
BI: Behavioural intention
CB: Challenge-skill-balance
CG: Clear goals
CoP: Centre of pressure
CT: Concentration at task
EE: Effort expectancy
FB: Facilitating conditions
FSS: Flow state scale questionnaire
HR: Heart rate
IREX®: GestureTek, Interactive Rehabilitation and Exercise System
LOS: Limits of stability
ML: Medial-lateral
NPRS: Numerical pain rating scale
OSI: Overall stability index
PE: Performance expectancy
PC: Paradox of control
RPE: Borg Rate of Perceived Exertion
SE: Self-efficacy
SI: Social influence
SMEQ: Subjective Mental Effort Questionnaire
TGB: Traditional gym-based exercise with no virtual stimuli
TT: Transformation of time
UF: Unambiguous feedback
UTAUT: The Unified Theory of Acceptance and Use of Technology questionnaire
VR: Virtual-reality
